# Niche Occupation Limits Adaptive Radiation in Experimental Microcosms

**DOI:** 10.1371/journal.pone.0000193

**Published:** 2007-02-07

**Authors:** Michael A. Brockhurst, Nick Colegrave, David J. Hodgson, Angus Buckling

**Affiliations:** 1 School of Biological Sciences, Biosciences Building, University of Liverpool, Crown Street, Liverpool, United Kingdom; 2 Institute of Evolutionary Biology, School of Biological Sciences, University of Edinburgh, Edinburgh, United Kingdom; 3 Centre for Ecology and Conservation, School of Biological and Chemical Sciences, University of Exeter in Cornwall, Penryn, United Kingdom; 4 Department of Zoology, University of Oxford, Oxford, United Kingdom; University of Chicago, United States of America

## Abstract

Adaptive radiations have played a key role in the evolution of biological diversity. The breadth of adaptive radiation in an invading lineage is likely to be influenced by the availability of ecological niches, which will be determined to some extent by the diversity of the resident community. High resident diversity may result in existing ecological niches being filled, inhibiting subsequent adaptive radiation. Conversely, high resident diversity could result in the creation of novel ecological niches or an increase in within niche competition driving niche partitioning, thus promoting subsequent diversification. We tested the role of resident diversity on adaptive radiations in experimental populations of the bacterium *Pseudomonas fluorescens* that readily diversify into a range of niche specialists when grown in a heterogeneous environment. We allowed an undiversified strain to invade resident communities that varied in the number of niche specialists. The breadth of adaptive radiation attainable by an invading lineage decreased with increasing niche occupation of the resident community. Our results highlight the importance of niche occupation as a constraint on adaptive radiation.

## Introduction

Adaptive radiations are thought to play an important role in the evolution of biodiversity. A single colonizing lineage will diversify into coexisting niche specialists if there are unoccupied ecological niches and strong competition within the coloniser's niche [1]. While competition is the key factor driving selection, it is the availability of multiple ecological niches in heterogeneous environments that causes selection to be divergent leading to adaptive radiation [1], [2], [3]. The diversity of the resident community is therefore likely to play a key role in determining the extent of diversification, although whether high resident diversity will inhibit or promote subsequent diversification is unclear. First, high resident diversity may result in physical ecological niches being more fully occupied, hence inhibiting diversification of an invading lineage. Second, increasing the number of resident species may increase the complexity of biotic interactions thereby creating novel niches or driving finer scale niche-partitioning [1], [4], [5], [6]. A recent study observed higher rates of endemic speciation (i.e., diversification) on islands containing more species [6], supporting the latter prediction. However, in such correlation studies, it is impossible to rule out the possibility that the pattern was the result of other, unmeasured, covariates.

Here, we address whether resident niche diversity has a net positive or negative effect on the extent of diversification in an invading lineage in a highly controlled microbial system. In heterogeneous environments (static microcosms or vials containing growth media) populations of the bacterium *P. fluorescens* rapidly diversify into three spatial niche specialist classes readily distinguished by heritable colony morphology and niche occupation [2]. Smooths (SM) resemble ancestral colonies and inhabit the liquid; wrinkly-spreaders (WS) have rough colonies and form a mat at the air-liquid interface; fuzzy-spreaders (FS) have diffuse colonies and inhabit the bottom of the vials. It should be noted that a diverse range of colony morphology variants has been observed within each of these spatial niche specialist classes. Thus multiple morphotypes may coexist within spatial niches in single populations [2], [7], [8], [9], and different morphotypes may evolve to occupy a given spatial niche in different populations [10], [11]. Molecular studies suggest that different niche specialist morphotypes are likely to result from different mutational events [12].

Competition for resources is responsible for the origin and maintenance of diversity, as demonstrated by the fact that rare spatial niche specialists have a fitness advantage due to less intense competition within their spatial niche (negative frequency dependent selection [13]). Therefore, resident spatial niche specialist morphotypes may be expected to reduce selection for diversification of an invading lineage into a spatial niche by increasing the level of competition therein. However, as noted above, diversity has been observed to evolve *within* each spatial niche: multiple variants of each morphoytpe are commonly present within evolved populations. This diversity arises to some extent through resource partitioning within each of the spatial niches and is maintained by frequency dependent selection [7], [14]. Thus by exerting within niche resource competition, resident niche specialist morphotypes may increase selection for divergence *within* each spatial niche (i.e., niche-partitioning). While the creation of novel “biotic” niches has been observed in laboratory bacterial populations (notable examples include the evolution of cross-feeding mechanisms in *Escherichia coli* [15], and the evolution of non-siderophore-producing “cheats” in *Pseudomonas aeruginosa* [16]), such mechanisms have not previously been reported to maintain diversity in *P. fluorescens* populations. As such, it is plausible that through modification of competitive interactions within and between niches rather than creation of novel niches, resident niche occupation could potentially promote or inhibit diversification of an invading lineage.

We isolated six clones within each spatial niche specialist class (SM, WS and FS) and constructed communities that varied in the number of the primary niche specialists (0, 1, 2, or 3) and total morphotypic diversity (0, 1, 2, 3 or 6 morphotypes), following a similar experimental design to Hodgson *et al.* 2002 (Reference 19; see also table 1 for details of treatments). We then allowed an ancestral lineage (i.e., with SM-like colony morphology and niche preference) to invade each community from rare (i.e., at a ratio of 1∶100 of invader to residents respectively) and measured the diversity of the invading lineage after three days of static incubation. We were able to distinguish the invading lineage from the residents by plating on selective media. The ancestral lineage was used as the invader because evolved morphotypes display greatly reduced potential for diversification [17]. Crucially, the resident niche specialist clones were each isolated from an independent, parallel adaptive radiation and were thus phenotypically (i.e., colony morphology) and genotypically distinct [12]. Previous work further suggests that different morphotypes within spatial niches will be ecologically distinct in terms of resource use [14], [18]. We therefore, in line with previous studies using this experimental system considered these distinct asexual lineages to be equivalent to species (for example see [2], [19]).

**Table 1:** Details of constructed mixtures and their morphotypic and spatial niche specialist class diversities.

Resident community	Morphotypic diversity	Spatial niche diversity
s1s6	2	1
s2s4	2	1
s5s3	2	1
f3f5	2	1
f1f2	2	1
f4f6	2	1
w3w6	2	1
w5w4	2	1
w1w2	2	1
s6w4	2	2
s4w2	2	2
w6f5	2	2
w5f4	2	2
s3f1	2	2
s1f2	2	2
s2w1	2	2
s5f3	2	2
f6w3	2	2
s5s3s1	3	1
s2s6s4	3	1
w1w2w3	3	1
w4w5w6	3	1
f3f4f6	3	1
f1f2f5	3	1
s1s6w1	3	2
s4s5f1	3	2
w2w3f3	3	2
w4w5s3	3	2
f4f5s2	3	2
f2f6w6	3	2
s2f3w5	3	3
s4f2w4	3	3
s3f6w6	3	3
s5f4w2	3	3
s6f5w3	3	3
s1f1w1	3	3
s1s2s3s4s5s6	6	1
w1w2w3w4w5w6	6	1
f1f2f3f4f5f6	6	1
s2s3s6w1w5w6	6	2
w2w3w4f3f4f5	6	2
s1s4s5f1f2f6	6	2
s1s6f1f4w4w5	6	3
s3s5f2f6w1w3	6	3
s2s4f3f5w2w6	6	3

The 18 monocultures are not shown. s = smooth; f = fuzzy spreader; w = wrinkly spreader.

## Results and Discussion

**Figure 1:**
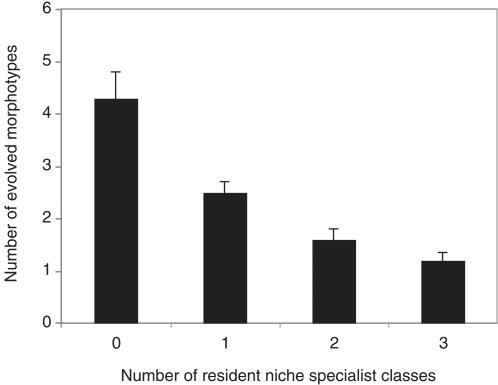
Diversification of an initially rare invading lineage as a function of the number of resident niche specialists. Bars show mean (+SEM) number of novel morphologically distinct morphotypes; this value does not include the invading ancestral genotype.

Diversification of the invading lineage (as measured by the number of novel, distinct morphotypes) decreased with increasing niche occupation by resident spatial niche specialists (Fig. 1; *F*_1,66_ = 20.75, *P*<0.001). However, there was no significant additional effect of within-spatial-niche morphotypic diversity of the resident community on invading lineage diversification (*F*_1,66_ = 0.24, *P*>0.1). This suggests that within a given spatial niche a single occupying morphotype is as effective as multiple occupying morphotypes at inhibiting an invader's diversification. This result is surprising given that resource partitioning can occur within each spatial niche, as evidenced by the occurrence of negative frequency dependent selection of coevolved morphotypes [7], [14]. Each of the three different niche specialist classes caused a significant reduction in the diversification of the invading lineage when they were resident, but WS (*F*_1,65_ = 78, *P*<0.001) had a much more pronounced effect than either SM (*F*_1,65_ = 3.9, *P* = 0.05) or FS (*F*_1,65_ = 5.03, *P* = 0.03). These data extend findings from purely ecological studies in a range of systems (including this system, see reference 17), where increased diversity tends to confer greater community resistance to invasion [20]. Here, we show that more functionally replete communities are likely to be better able to resist evolutionary invasion of niche space by a diversifying invading lineage. Note that the same qualitative results are obtained when the ‘zero resident diversity’ treatment is excluded from the analysis.

These results strongly suggest that resident niche occupation is most likely to inhibit, rather than promote, diversification of an invading lineage. However, the patterns of inhibition are relatively complex, even in this simple experimental system. Specifically, there is not a one to one relationship between resident niche occupation and whether or not the invading lineage diversifies to occupy that niche (Table 2). Resident WS significantly inhibited evolution of WS: invading lineages evolved novel WS in 30 out of 36 communities that lacked resident WS, but never when resident WS were present (Fisher's exact test: *P*<0.001). However, resident WS also significantly inhibited evolution of FS: novel FS evolved in 7 out of 36 communities that lacked resident WS, but never when WS was resident (Fisher's exact test: *P* = 0.005). The presence of resident WS did not significantly influence the establishment of novel SM morphotypes (*P*>0.1). Resident FS entirely prevented the establishment of FS in all cases, compared with 7 out of 36 in the absence of FS residents (Fisher's exact test: *P* = 0.005). Resident FS did not significantly inhibit the evolution of SM (*P*<0.05; but, this is not significant after application of Bonferroni correction for multiple comparisons), nor WS (*P*>0.1). SM did not appear to affect the establishment of any particular niche specialist (*P*>0.1 for SM, WS and FS), but had an overall slight inhibitory effect on diversification, as outlined in the first paragraph of this section. Note that where diversification occurred, no more than one new SM morphotype (in addition to the invading ancestor) or one FS morphotype was detected per community. By contrast, up to 4 WS evolved morphotypes were detected per community.

**Table 2:** Populations containing evolved spatial niche specialists as a function of the presence or absence of resident spatial niche specialists.

	RESIDENT SPATIAL NICHE SPECIALIST					
	WS		SM		FS	
EVOLVED SPATIAL NICHE SPECIALIST	PRESENT	ABSENT	PRESENT	ABSENT	PRESENT	ABSENT
WS	0/33	30/36	14/33	17/36	14/33	16/36
SM	10/33	10/36	10/33	17/36	8/33	18/36
FS	0/33	7/36	4/33	3/36	0/33	7/36

That within-spatial niche (genotypic) diversity did not affect invader diversification has important consequences for total community diversity after an invader has diversified, particularly with respect to the contribution of WS to total diversity. In the absence of a WS resident, the invading lineage produced an average of 1.7 novel WS morphotypes, whereas in the presence of a single WS, no novel WS morphotypes were detected. As such, total community diversity was greatly reduced by the presence of a single WS resident (mean total number of morphotypes: no residents = 5.3; 1 WS resident = 2.3; *n* = 12, *t* = 5.6, *P* = 0.001), which itself underwent no detectable diversification during the experiment. These data show that although fitness tradeoffs between niches can result in the maintenance of diversity [2], they are not necessarily sufficient for the initial establishment of diversity.

It is important to emphasise the limitations of this study. First, we are only addressing the initial bursts of adaptive radiations. A longer-term study would be extremely problematic to carry out with this system because of changes in resident composition through time (although for a different approach see [21], [22]), but might reveal different patterns. For example, bacterial diversity has been shown to increase over much longer time scales, as a result, for example, of the evolution of cross-feeding mechanisms [15]. However, recent theory and data suggest that the most diversification will occur in the early stages of a population entering a new habitat [17], [23], when organisms are poorly adapted. Second, this is a simple ecosystem with only a single trophic level. In more complex ecosystems [6], [24], diversity could well open up new ecological niches, most obviously with respect to parasitism and predation.

The importance of vacant niches (or niche saturation) has been implicated in many historical patterns of diversification. For example, the rise of placental mammals has been suggested to have been driven by the sudden existence of niches, vacated by the extinction of large reptiles at the end of the Cretaceous [1]. Our results highlight the importance of niche occupation in determining the breadth of adaptive radiation attainable by an invading lineage. Our findings along with those of other studies (for example [10], [17], [25]) highlight that constraints on adaptive radiation are likely to play a central role in determining why some communities harbour more biodiversity than others.

## Methods

18 evolved morphotypes (6 SM, 6 WS and 6 FS) of *P. fluorescens* SBW25 *panB*, a mutant strain that requires an exogenous source of pantothenic acid [2], were obtained [19]. We used the *panB* morphotypes to construct mixtures that varied in total morphotypic diversity (0, 1, 2, 3 & 6 morphotypes) and the number of occupied niches (‘functional diversity’; 0–3). Each combination of morphotypic and functional group diversity was replicated to the extent that, within each combination of genotypic and functional diversity, all communities were independent from each other, and all morphotypes were equally represented. Within these constraints, the choice of particular morphotype combinations was random. This resulted in a total of 45 mixtures [19], plus the 18 monocultures and 6 replicate “no resident” treatments. Details of each community and samples sizes for the different treatment combinations are shown in Table 1.

Cultures were established by first growing morphotypes for 18 hours in microcosms (30 ml glass universals with loose plastic lids, containing 6ml of King's Medium B (KB) supplemented with 0.0024% pantothenic acid) at 28°C in a 200 rpm orbital shaker, an then inoculating into new microcosms at equal densities, with total mixture density 100-fold less than the maximum afforded by the microcosms. The invading genotype, ancestral *P. fluorescens* SBW25 morphotype SM, was simultaneously inoculated with the residents, but at 100-fold lower density. Simultaneous inoculation of residents and invaders was necessary to prevent disturbance of the microcosms during the growth phase. Populations were then propagated in a static incubator at 28°C for 3 days. We measured diversity as the total number of morphologically distinct invader morphotypes, as determined from 100 random colonies [8], [10] plated onto vitamin-free casein agar plates that prevented growth of the resident population. Counts were square-root-transformed, and analysed as General Linear Models (Minitab®), with (i) morphological and functional diversity of the resident community as covariates, (ii) the presence or absence of each of WS, FS and SM were fitted as factors. Significance of terms was determined by removal from maximal models [26].
